# Statistical process control for performance monitoring and continuous quality assurance of deep learning segmentations in radiotherapy

**DOI:** 10.1016/j.phro.2025.100873

**Published:** 2025-11-26

**Authors:** Niels van Acht, Dave van Gruijthuijsen, Johanna Bluemink, Els Hagelaar, Coen Hurkmans

**Affiliations:** a Department of Radiation Oncology, Catharina Hospital, Eindhoven, the Netherlands; b Department of Electrical Engineering, Eindhoven University of Technology, Eindhoven, the Netherlands; c Department of Applied Physics and Science Education, Eindhoven University of Technology, Eindhoven, the Netherlands

**Keywords:** Continuous quality assurance, Statistical process control, Deep learning segmentation, Radiotherapy planning, Deep learning monitoring

## Abstract

**Background and purpose:**

After clinical implementation of deep learning segmentation (DLS) models it is highly recommended to perform routine quality assurance (QA). Through forthcoming regulations of the EU AI Act state that the output of any DLS model needs to be logged, enabling continuous QA (CQA) to monitor DLS performance and introduce alarms. Therefore, the goal was to implement a CQA framework for DLS in radiotherapy.

**Materials and methods:**

The direct output of the DLS models and the clinically approved delineations (CS) were automatically exported, after which geometric metrics were calculated. For each combination between a region of interest (ROI) and metric, a target, lower and upper control limit were determined, based on statistical process control (SPC). Adapted versions of the first three Nelson rules were used for outlier identification and detection of trend shifts and drifts.

**Results:**

In the first six months, 545 DLS and corresponding CS RT structure files were logged containing 3093 ROIs. From these ROIs, 3.0 % was automatically reported as outlier. Data from 5 patients, which anatomy was deemed interesting, was saved for potential model re-training. Four trend shifts identified a performance drop for the humerus due to unexpected changes. Twelve other trend shifts and one trend drift were detected, causing only temporary deviations.

**Conclusions:**

A CQA framework for DLS was successfully implemented using SPC and adapted Nelson rules to automatically report outliers and trend shifts, which led to automatic detection of such deviations.

## Highlights

• Deep learning and clinical segmentations are logged in real-time.
• A new quality assurance framework for continuous monitoring.
• Open-source framework for accessible and institution-independent implementation.
• First study to apply statistical process control and Nelson rules in radiotherapy.
• In six months detected 93 outliers, 16 trend shifts and 1 trend drift.

## Graphical abstract

**Figure:**
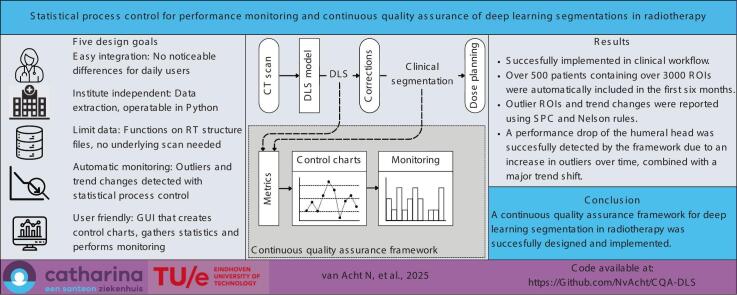

## 1 Introduction

In recent years, deep learning segmentation (DLS) has become commercially available for radiotherapy in software products such as treatment planning systems (TPS) [1]. Using DLS models it is now possible to automatically delineate organs at risk (OARs) and some target volumes (TVs). This eliminates the need for radiotherapy technicians (RTTs) and radiotherapy oncologists (ROs) to delineate images from scratch, resulting in a more efficient, faster, and consistent workflow [[2], [3], [4], [5]].

Although medical device regulation approval has already been received for commercially available DLS models, it is still necessary to commission the models at each institution before implementation [6]. During commissioning, the first step is to investigate whether or not the intended use of the model is in line with the current radiotherapy workflow. If it is detected that certain parts of the workflow are not in line with the intended use the DLS models should in principle not be clinically approved for that workflow. The next step is a quantitative analysis. A quantitative analysis uses geometric metrics to compare a DL segmentation to a gold standard segmentation. Examples of metrics for three-dimensional segmentations are the volumetric Dice similarity coefficient (VDSC), the surface Dice similarity coefficient (SDSC), the Hausdorff distance (HD), and the added path length (APL) [[7], [8], [9], [10], [11]]. In addition to a quantitative analysis a qualitative analysis is essential to evaluate the clinical acceptability of the DLS models [2,3,6,12]. This qualitative analysis involves end-users scoring the DLS usability and measuring the time difference between the current and proposed DLS methods. After investigating the intended use and performing both analyses one can decide to clinically implement DLS in the radiotherapy workflow [6].

After implementation it is still highly recommended, and soon to be finalised into a code of practice by the EU, to perform routine quality assurance (QA) on a group of test patients [6,[13], [14], [15]]. The FUTURE-AI guideline has been established through consensus based on six guiding principles [16]. One of these principles is traceability, which states that medical AI tools should be developed together with mechanisms for documenting and monitoring. Therefore, it is mentioned that research in this area should investigate possibilities for: continuous monitoring and quality control, identification of data or concept drifts and biases, and time series statistics and visualisation.

Despite multiple articles mentioning the importance of quality control or assurance towards effective and reliable machine learning (ML), few articles mention a concise approach [16,17]. Sahiner et al. [18] mentioned how ML output performance monitoring can provide a direct measure of performance decay. They highlighted the potential of statistical process control (SPC) for drift detection without the provision of new data to support this. Similarly, Xiao et al. [19] stated that radiotherapy research should intensify on SPC theories and methods for radiotherapy QA. Muralidharan et al. [20] discussed the possibility to further optimise the assessment of control charts in the medical domain by using Nelson rules, first proposed by Nelson et al. [21]. They discussed that, for medical purposes, the first three rules could limit the amount of false alarms as they are designed to detect outliers, trend shifts and trend drifts.

Taking into account the forthcoming regulations from the AI Act and FUTURE-AI’s proposals for research, the goal of this article was to design a continuous quality assurance (CQA) framework for deep learning segmentation in radiotherapy. It was proposed that by implementing SPC in combination with Nelson rules, the framework should be able to automatically detect outliers and trend shifts and trend drifts. After the design process, the CQA framework was implemented in the clinical workflow and evaluated with real-time clinical data.

## 2 Materials and methods

### 2.1 Commissioning

Since November 2022, over time, a total of 33 ROIs were commissioned for clinical use from which the quantitative and qualitative analysis can be found in Supplementary Table S1. Of the 33 ROIs, 12 ROIs were commissioned with a DLS model trained in-house by Bakx et al. [12] and were therefore not included in the current commissioning dataset. The remaining commissioned ROIs were part of the embedded DLS model of TPS RayStation from RaySearch laboratories AB. In this research, RayStation version 12A was used up to 15 weeks after the start of logging. Thereafter, version 2024B was used.

### 2.2 Setup of continuous QA

Five goals were established for the CQA framework, explained in Table 1. The final goal in Table 1 was to develop a system for automatic monitoring of the DLS model using outlier detection and trend shift and trend drift identification. In this context, an outlier refers not to an out-of-distribution patient, but to a ROI with an adjustment differing from similar ROIs. Likewise, an SPC trend shift and SPC drift refers to a noticeable consecutive change in adjustments per ROI, rather than a change in the entire dataset.

**Table 1:** Table showing the design goals used to create the CQA framework with their justification.

Design goal	Justification
Seamless Integration	Ensured that the system did not disrupt the end-user workflow, as any noticeable impact on speed or the introduction of additional tasks would reduce user satisfaction.
TPS Independence	Ensuring TPS independence was crucial for the system to be easily deployable across various institutions, thereby increasing flexibility and scalability.
Minimise Additional Data Storage	Reducing data storage requirements was necessary to avoid unnecessary duplication, as all patient data had already been retained since the implementation of RayStation.
Graphical User Interface (GUI)	A user-friendly GUI was essential to enable clinicians to visualise relevant data quickly and accurately.
Automatic Outlier Detection & Trend Identification to monitor model	This was essential for detecting unexpected changes in the workflow and identifying potential automation biases.

**Fig. 1:**
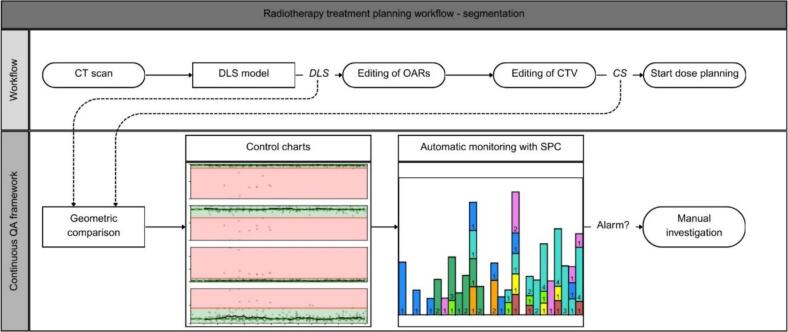
Flowchart of the segmentation part of the radiotherapy treatment planning workflow. The top part of the charts shows how and where the data required for CQA of DLS is created and saved. Here, DLS and CS refer to the deep learning segmentation and the clinical segmentation, the stage in which the segmentation was saved. The bottom part shows the CQA framework. Here geometric comparison represents the data storage and the geometric evaluation. This evaluation is used to visualise the data over time in control charts. Automatic monitoring with SPC refers to the implemented statistical process control and the adapted Nelson rules that can induce an alarm. When an alarm was given, manual investigation to find the origin of this alarm was required.

In line with these design goals RT structure DICOM files were exported twice during radiotherapy planning, as illustrated in Fig. 1. The first export occurred immediately after DL segmentation ROIs were generated, with no modifications (DLS). The second export occurred after the plan was clinically approved by the RO, referred to as clinical segmentation (CS). The first RT structure file was automatically exported on the 28th of August of 2024. In the first six months, this resulted into 545 saved DLS and corresponding CS RT structure files containing 3093 ROIs. As the DLS and CS originated from the same scan, the contour points corresponded to identical coordinates in the frame of reference, ensuring the underlying scan was no longer necessary for evaluation in Python. This reduced the memory requirement per patient to approximately 3 MB.

A Python (version 3.8) analysis script was developed to calculate the VDSC, SDSC with 3 mm tolerance, HD95, and APL for all ROIs, saving the results in a patient-specific NumPy file. A GUI was created to allow visualisation of the results in the same Python version.

Automatic outlier, trend shift and trend drift detection was achieved using SPC parameters, with target values and lower (LCL) and upper control limits (UCL) derived from the system output. Since data from controlled environments, such as commissioning datasets, did not correctly represent real-time data, the SPC parameters were based on measurements from the CQA dataset. As per standard SPC practice, the target was set to the mean. The LCL and UCL were initially set at three standard deviations (σ) below and above the target. However, for increased sensitivity, 2σ was chosen instead. Data normality was assessed using the Shapiro-Wilk test. If the data was not normally distributed, the median was used as the target, with the 2.3rd and 97.7th percentiles as the LCL and UCL, respectively, representing the theoretical boundaries for a normally distributed dataset. These SPC parameters, along with a set of established rules, enabled automatic detection of outliers and trend shifts and trend drifts. From Nelson's eight rules, described in Supplementary Table S2, the first three rules were incorporated into the CQA framework, with slight modifications to better suit this use case (see Supplementary material A). A summary of these rules is provided in Table 2, with a visual representation in Supplementary Fig. S1. The framework is available at https://www.github.com/NvAcht/CQA-DLS.

**Table 2:** Table showing the purpose, original detection method and adapted detection method of the three Nelson rules used [20,21]. For the application in the CQA framework the rules were adapted to better fit its purpose, further explained in the Supplementary material A.

	Purpose	Original detection method	Adapted detection method
**Rule 1**	Detect outliers	One data point outside the control limits	For at least three of the four geometric metrics, one data point outside the control limits
**Rule 2**	Detect a trend shift	Nine consecutive data points on one side of the target	For at least one of the four geometric metrics, nine consecutive data points on one side of the target ± 0.5 % tolerance.
**Rule 3**	Detect a trend drift	Six consecutive data points either increasing or decreasing	For at least one of the four geometric metrics, six consecutive data points either increasing or decreasing

## 3 Results

Although not statistically significant, the difference in Table 3 showed that consistently less adjustments were made to the DLS in clinical practice than during commissioning. Statistically significant differences were tested with a student *t*-test if both CQA and commissioning data were normally distributed. For any other cases the Mann-Whitney *U* test was used.

**Table 3:** Table with the VDSC, SDSC at 3 mm tolerance, HD95, and APL determined during the CQA. N represents the number of times that ROI is present in the CQA dataset. The percentage that the DLS is accepted without correction is represented by % AWC. The median and IQR are shown instead of mean and standard deviation for the metrics as most of the data does not follow a normal distribution. Where possible, the difference in the median between the CQA data and the commissioning data in Table S1 is represented. Statistical testing showed that none of the differences were statistically significant (p-value < 0.05). Esophagus and Esophagus_Upper refer to the same ROI in the DLS models, but originate from different protocols and are thus named differently. The same holds for SpinalCanal and Spinal_canal.

ROI	N	% AWC	VDSC [-]				SDSC 3 mm [-]				HD95 [mm]				APL [N_voxels_]		
			Median	Difference	IQR	Min-Max	Median	Difference	IQR	Min-Max	Median	Difference	IQR	Min-Max	Median	IQR	Min-Max
**Bladder**	9	22.2	1.00	0.04	0.01	0.99–1.00	1.00	0.01	0.01	0.96–1.00	0.0	−2.8	1.2	0.0–3.0	442	614	0–3268
**Brain**	53	49.1	1.00	0.01	0.00	0.99–1.00	1.00	0.00	0.00	0.98–1.00	0.0	−1.2	0.0	0.0–1.2	109	1229	0–4019
**Brainstem**	53	1.9	0.89	0.00	0.05	0.00–1.00	0.97	0.00	0.04	0.00–1.00	3.0	0.0	0.5	0.0–94.2	1253	502	0–3458
**Breast_CL**	170	47.1	1.00	−	0.01	0.00–1.00	1.00	−	0.03	0.00–1.00	0.0	−	3.0	0.0–222.0	375	2092	0–38848
**Breast_IL**	37	21.6	0.98	−	0.03	0.00–1.00	0.99	−	0.07	0.00–1.00	1.2	1.2	6.1	0.0–221.1	2005	6572	0–39146
**CTVimn**	7	0.0	0.88	−	0.07	0.71–0.96	0.95	−	0.04	0.86–0.98	3.5	−	2.1	2.3–5.8	790	250	210–2103
**CTVn1**	78	0.0	0.85	−	0.12	0.19–1.00	0.78	−	0.21	0.26–1.00	11.8	−	9.6	0.0–57.0	2946	2598	12–9818
**CTVn2**	82	1.2	0.89	−	0.11	0.57–1.00	0.93	−	0.11	0.68–1.00	6.0	−	9.4	0.0–43.3	546	679	0–3228
**CTVn3**	63	0.0	0.89	−	0.09	0.23–1.00	0.93	−	0.11	0.20–1.00	5.2	−	3.1	0.0–33.7	403	261	0–4247
**CTVn4**	62	0.0	0.58	−	0.23	0.26–0.89	0.65	−	0.18	0.33–0.98	12	−	6.5	3.0–33.5	406	252	122–5472
**CTVp**	198	0.5	0.96	−	0.04	0.00–1.00	0.93	−	0.10	0.00–1.00	6.0	−	9.0	0.0–292.3	7892	6273	0–56720
**Esophagus**	152	30.3	0.99	0.19	0.05	0.17–1.00	1.00	0.06	0.02	0.28–1.00	0.0	−6.0	3.0	0.0–123.4	126	464	0–21294
**Esophagus_Upper**	17	23.5	0.99	0.19	0.03	0.84–1.00	0.99	0.05	0.02	0.89–1.00	0.0	−6.0	3.0	0.0–13.9	97	173	0–1575
**Femur_Head_L**	10	90.0	1.00	0.14	0.00	1.00–1.00	1.00	0.15	0.00	1.00–1.00	0.0	−26.7	0.0	0.0–0.0	0	0	0–151
**Femur_Head_R**	10	100.0	1.00	0.11	0.00	1.00–1.00	1.00	0.11	0.00	1.00–1.00	0.0	−16.5	0.0	0.0–0.0	0	0	0–0
**Heart**	437	6.6	0.97	0.03	0.03	0.00–1.00	0.90	0.03	0.05	0.00–1.00	9.0	0.8	6.0	0.0–268.2	4932	3554	0–37966
**Humerus**	67	38.8	0.93	−	0.58	0.00–1.00	0.96	−	0.59	0.00–1.00	3.7	−	14.8	0.0–116.3	983	3175	0–4456
**Kidney_L**	44	79.6	1.00	0.05	0.00	0.96–1.00	1.00	0.03	0.00	0.95–1.00	0.0	−2.4	0.0	0.0–6.0	0	0	0–2066
**Kidney_R**	44	75.0	1.00	0.07	0.00	0.95–1.00	1.00	0.07	0.00	0.93–1.00	0.0	−3.5	0.0	0.0–4.7	0	0	0–2701
**Lens_L**	52	92.3	1.00	0.24	0.00	0.23–1.00	1.00	0.00	0.00	0.87–1.00	0.0	−1.1	0.0	0.0–3.4	0	0	0–39
**Lens_R**	52	88.5	1.00	0.19	0.00	0.47–1.00	1.00	0.00	0.00	0.96–1.00	0.0	−1.1	0.0	0.0–3.5	0	0	0–23
**Liver**	43	69.8	1.00	0.02	0.00	0.98–1.00	1.00	0.02	0.00	0.93–1.00	0.0	−1.4	0.0	0.0–8.1	0	84	0–11983
**Lung_L**	463	79.1	1.00	0.05	0.00	0.84–1.00	1.00	0.14	0.00	0.79–1.00	0.0	−7.8	0.0	0.0–46.5	0	0	0–25787
**Lung_R**	463	83.6	1.00	0.04	0.00	0.79–1.00	1.00	0.08	0.00	0.72–1.00	0.0	−5.0	0.0	0.0–111.7	0	0	0–24986
**OpticNrv_L**	53	1.9	0.85	0.15	0.12	0.63–1.00	0.94	0.03	0.08	0.74–1.00	4.8	−4.5	4.5	0.0–19.2	1	2	0–59
**OpticNrv_R**	53	1.9	0.85	0.21	0.09	0.58–1.00	0.92	0.02	0.08	0.75–1.00	6.0	−2.6	6.1	0.0–17.9	4	9	0–65
**Pituitary**	50	26.0	0.77	0.02	0.34	0.33–1.00	1.00	0.00	0.01	0.82–1.00	2.3	0.6	2.7	0.0–5.6	51	100	0–276
**Rectum**	8	0.0	0.96	0.09	0.07	0.82–1.00	0.96	0.09	0.07	0.76–1.00	4.5	−3.5	11.2	0.0–22.6	1292	2188	184–6216
**Spinal_canal**	45	91.1	1.00	0.18	0.00	0.99–1.00	1.00	0.11	0.00	1.00–1.00	0.0	−70.1	0.0	0.0–0.0	0	0	0–456
**SpinalCanal**	112	87.5	1.00	0.18	0.00	0.86–1.00	1.00	0.11	0.00	0.85–1.00	0.0	−70.1	0.0	0.0–81.1	0	0	0–3600
**Spleen**	44	68.2	1.00	0.08	0.01	0.66–1.00	1.00	0.03	0.01	0.67–1.00	0.0	−3.0	0.3	0.0–27.5	0	302	0–3783
**Glnd_Thyroid**	60	50.0	1.00	−	0.02	0.35–1.00	1.00	−	0.01	0.35–1.00	0.0	−	1.2	0.0–29.7	0	83	0–9308

Rule 1 detected 88 outliers with larger adjustments and 5 with less adjustments. This corresponded to 3.0 % of the total ROIs being detected as outliers. These 93 outliers were manually investigated and from them 12 were found to be human errors.

**Fig. 2:**
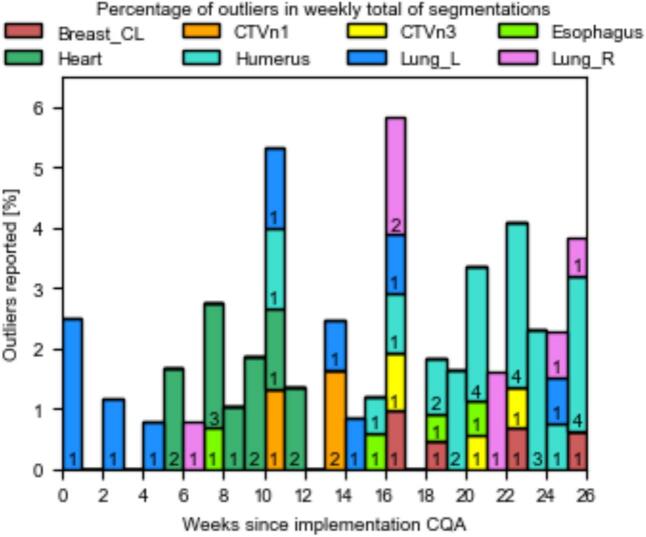
Bar chart showing the percentage of outliers detected by the first adapted Nelson rule within the weekly total of ROIs. Only ROIs that have been reported as outliers more than twice are included for easier visualisation. Additionally to the percentage, the absolute number per ROI per week is shown as well in the corresponding bar.

**Fig. 3:**
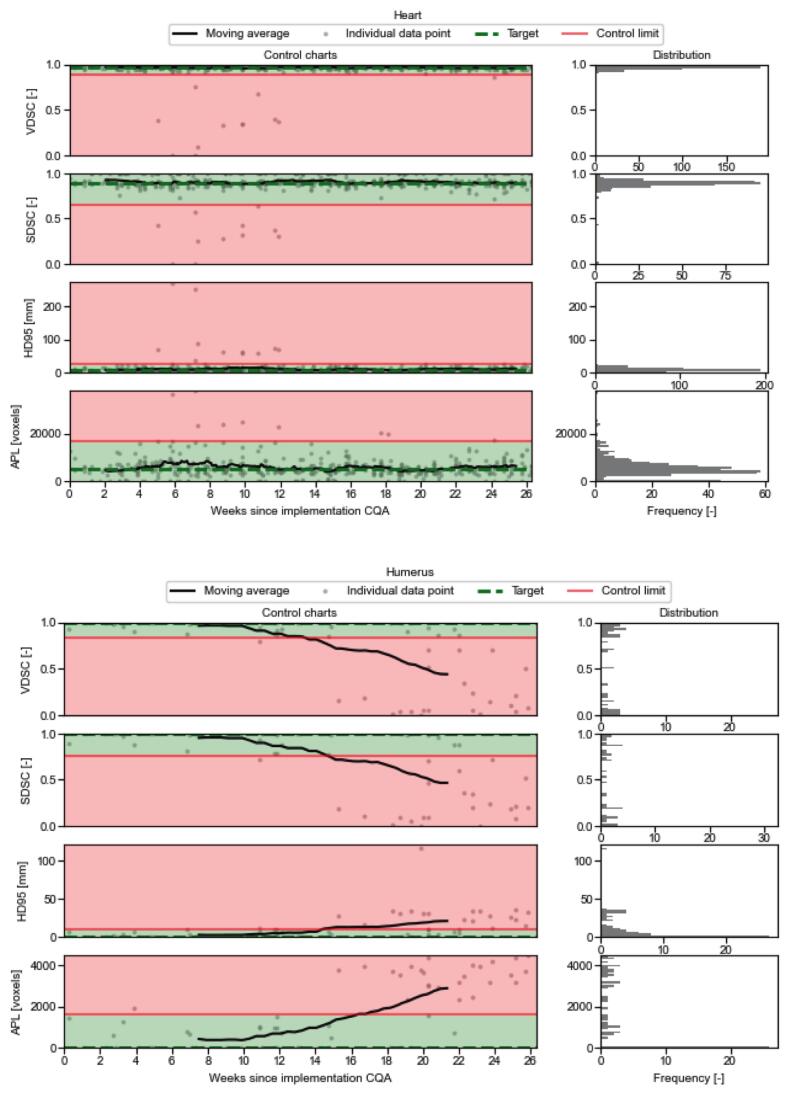
Control charts automatically created by the GUI, showing the VDSC, SDSC at 3 mm tolerance, HD95 and APL over time for the heart (A) and humerus (B) together with the distribution. The control charts use SPC to show the target, LCL, UCL, together with the boundaries they create. Individual data points and a moving average are also plotted. This moving average is determined by taking the average of the closest 30 neighbouring datapoints.

When looking at the frequency of ROIs being detected as outliers over time in Fig. 2, it could be seen that since the upgrade to RayStation version 2024B the amount of reported heart outliers decreased and humerus outliers increased. For the heart, this decrease did not result in a shift in the trend (Fig. 3A). However, for the humerus the increase did result in a shift in the trend. For RayStation version 12A, no trend shifts were reported for the humerus, whereas 4 trend shifts were reported for version 2024B. This resulted in a shift of the moving average outside the control limits (Fig. 3B). This did not occur for the other 12 reported trend shifts, as they only caused a temporal deviation from the target within the control limits. Only one trend drift was detected, where the APL increased for the brainstem 7 times in a row. This drift did not result in a deviation of the moving average.

## 4 Discussion

The goal to develop a system that continuously monitors the performances of the DLS models, following the rules of the AI Act and FUTURE-AI guidelines, was achieved by implementing all five set design goals. By making use of SPC and adapting the Nelson rules, 3.0 % of ROIs were reported as outliers. Although this was lower than the theoretical 4.6 %, which would result from a large number of normal distributed data, it was seen as an acceptable sensitivity for a continuous approach. This is especially important given that it showed sensitivity by detecting a higher percentage of larger adjustments outliers than the theoretical 2.3 %.

Although not statistically significant, an interesting result was the consistency that the DLS were adjusted less in the CQA dataset compared to commissioning dataset. This could indicate an automation bias within the institute. However, an automation bias is known to arise over time after an overreliance on the ML system is established, similar to a trend drift [18,20]. More likely this difference had to do with the nature of the task. During commissioning the geometric metrics served as baseline measurement of the DLS models. Therefore the end user was required to adjust the DLS to what they saw as the ground truth. Although they were instructed to mimic a clinical setting, it was reasonable to assume that they took extra care in adjusting the DLS. Moreover, unpublished data within the institute showed that not all adjustments to the DLS were clinically relevant. Similarly, Mody et al. [22] found that there was low dose impact on using DLS for OARs over CS in head-and-neck radiotherapy. Therefore, end-users were instructed not to spend too much time altering structures in the clinical workflow that were known to not have a clinical impact. Although this was optimal for the clinical workflow, using this clinical data as gold standard for training or validation of a model would not be accurate.

During the manual review process, backups were created of five patients, each containing CT images, clinically approved structures, and the corresponding treatment plan and dose. These patients were selected to potentially discuss with the manufacturer for retraining due to interesting anatomical variations. Examples were: intestines filled with air close to the lungs, a seroma under the armpit, and fluid accumulation in the lungs. Although the primary objective of this framework was not to identify all anatomical variation, as human oversight remained present for every case, preserving these datasets for potential retraining might enhance the robustness of future models since it provides variation in the training set, similar to data augmentation [23].

Before CQA with SPC, the institute relied on less consistent QA and end-users to report performance drops. Although the current setup was not tested for sensitivity and specificity, with the integration of the CQA framework, a clinically significant performance drop of the humerus was detected automatically. Simultaneously, end-users recognised the same performance drop in their daily practice, supporting the outcome of the CQA framework. However, future work would be needed to examine the sensitivity and specificity to data outside the intended use and possible improvements.

This approach for CQA detected outliers retrospectively which enabled the detection of trend changes by monitoring outlier counts over time. However, one might also try to predict potential outliers prospectively. Jin et al. [24] showed how they were able to predict auto-segmentation quality based on a CT scan and the auto-segmentation. An approach similar to Jin et al. could be implemented to predict the potential geometric metrics. With these predicted metrics it can be checked whether the patient will contain an outlier according to the adapted Nelson rules, advising the end-users to be more alert for that specific ROI.

It was not possible to prevent end-users from using DLS models out of their intended use. Although this was not seen as a problem, as there are multiple humans-in-the-loop, in the future this might become a problem as the human-in-the-loop might shift to a later point in the workflow potentially increasing risks or necessitating extra quality checks [25].

The SPC parameters were determined at a fixed point in time and therefore will not vary over time. They were calculated for ROIs that occurred more than ten times in the CQA dataset. Xiao et al. [19] noted that, for individual control charts, a sample size of 300 would be required to ensure the performance of control limits, but most radiation therapy QA studies used a sample size of 30. This emphasised the need to further investigate the optimal sample size. Over time, it might be beneficial to update the SPC parameters for less commonly exported ROIs as their sample size increases. However, since a sample size of 10 was used during commissioning, 10 data points were considered sufficient as starting point. Additionally, updates to the control limits might be necessary after a known trend shift occurs.

As a starting point, the second and third adapted Nelson rule were deployed to detect a trend shift and trend drift within the entire department. Despite the successful detection of a trend shift for the humerus by the second rule, no other trend shifts or drifts that could be linked to an automation bias or dataset shift were detected. Roschewitz et al. [26] mentioned how covariate shifts, a dataset shift which can be induced by a change of a CT scanner, protocol or patient demographic, could affect AI performance. They further specified that covariate shifts might lead to clinical errors. Although clinical errors were not likely due to expert checks, the methods described can still help identify data shifts between training and real-time data. These were not implemented in the current CQA framework, but were seen as a potential addition for future versions. Roschewitz et al. further mentioned that prevalence shifts, induced by changes in the frequency of disease incidence, would contribute to a decrease in AI performance. However, for an AI model used during treatment planning this should not be present, as this type of shift would occur at diagnostics only.

Automation bias and a learning curve might be something more easily detected at a per-user-level, by the second and third adapted Nelson rule respectively. Therefore, it was suggested to consider keeping track of the metrics per end-user. This could especially be beneficial to investigate the learning curve of new users, to see whether they changed the number of adaptations after getting more acquainted with the approach and whether they operated within the inter-observer variation. The inter-observer variation had been used previously as minimum acceptance criteria for a new DLS model [2,6]. Therefore, if new users would not operate within this variation, this would be a trigger for further investigation.

Similar to analysing data at a per-user level, differentiating between tumour sites could yield different results as clinicians adjust the DLS based on more than just imaging [27]. In this study, all tumour sites were grouped for analysis, although the degree of adjustment varied per site, reflecting differences in clinical relevance. Currently, insufficient data prevented an exploration of these variations. To enable site-specific analysis in future work, treatment protocols have been incorporated into the structure file names.

After introducing a CQA framework for DLS, the next step would be to implement a similar approach for deep learning planning (DLP). De Kerf et al. [28] mentioned how the QA of a DLP model should compare dosimetric metrics, such as dose-volume histogram (DVH) parameters, and possibly combine it with geometric metrics. Next to saving the DVH of the DL plan, by saving the DVH of the clinically approved plan, a lot of insight on the performance of DL in every step of the radiotherapy workflow and the clinical impact of changes to DL segmentations could be gained. Here, SPC parameters could help to create clinical impact zones, possibly improving the approach of De Kerf et al. who used one standard deviation in local surface Dice and 1 Gy of absolute dose difference to create four clinical impact zones, the latter being a specification limit rather than a control limit as per SPC definitions. However, as the received dose is plan specific, comparing segmentations without dose is more generic.

As this CQA framework showed promising results in one institute, the next step would be to deploy it in multiple institutes to perform continuous inter-institutional monitoring. Since it solely required Python and commonly used RT structure files, other institutes should be able to do this without too much hinderance. However, one could argue that this should not be the responsibility of the deployer and should be provided by the supplier of the DL tools, as such QA is mandated by EU AI Act published in the Official Journal of the European Union [13,14]. Article 26 of the AI Act states that: “Deployers (the users) shall monitor the operation of the high-risk AI system.” and later on: “Deployers of high-risk AI systems shall keep the logs automatically generated by that high-risk AI system.” [15]. This would take significant amount of time of deployers to design, implement and maintain.

In conclusion, a CQA framework for DLS was successfully designed and implemented in the clinical workflow. Implementing SPC and adapting three Nelson rules, outliers and trend changes are now automatically reported. This has led to automatic detection of one major trend shift.

## CRediT authorship contribution statement

**Niels van Acht:** Methodology, Software, Validation, Formal analysis, Investigation, Writing – original draft, Writing – review & editing. **Dave van Gruijthuijsen:** Software, Data curation, Writing – review & editing. **Johanna Bluemink:** Data curation, Writing – review & editing. **Els Hagelaar:** Data curation, Writing – review & editing. **Coen Hurkmans:** Methodology, Writing – review & editing.

## Declaration of competing interest

Coen Hurkmans is an Editorial Board Member for this journal and was not involved in the editorial review or the decision to publish this article. The authors declare the following financial interests/personal relationships which may be considered as potential competing interests: A research grant from RaySearch Laboratories AB for Niels van Acht is gratefully acknowledged.
