# Supplementary material for: Statistical process control for performance monitoring and continuous quality assurance of deep learning segmentations in radiotherapy
Source: Phys Imaging Radiat Oncol. 2025 Nov 26;36:100873. doi: 10.1016/j.phro.2025.100873 (PMC12702409; doi:10.1016/j.phro.2025.100873)
Supplement: Supplementary Data 1 [file mmc1.pdf]

Appendix

Supplementary Table S1: Table with the VDSC, SDSC at 3 mm tolerance, HD95, t_{gain} and DL Score determined during the quantitative and qualitative analysis. t_{gain} is determined by taking the difference between the time required for the old method and the time required for the DLS method. The DL Score is given by the participants given the following rubric: 0: Not acceptable. Complete re-drawing needed. No time-gain. 1: Major corrections needed, but still usable. Minor time gain. 2: Minor corrections needed. Significant time gain. 3: Accepted without corrections. Significant time gain. Median and IQR are shown instead of mean and standard deviation as most of the data does not follow a normal distribution. The extrema, the minimum and maximum values, are given in the Extr column.

ROI	VDSC [-]			SDSC 3 mm [-]			HD95 [mm]			t_{gain} [s]			DL Score [-]		
	**Med**	**IQR**	**Extr**	**Med**	**IQR**	**Extr**	**Med**	**IQR**	**Extr**	**Med**	**IQR**	**Extr**	**Med**	**IQR**	**Extr**
**Anal_Canal**	0.71	0.11	0.48-0.86	0.83	0.26	0.49-0.94	6.1	4.4	3.3-12.0	74	65	-6-96	2	1.5	1-3
**Bladder**	0.96	0.02	0.94-0.97	0.99	0.03	0.95-1.0	2.8	1.3	1.2-3.2	142	90	69-272	3	0.1	1-3
**Brain**	0.99	0.00	0.98-0.99	1.00	0.01	0.98-1.0	1.2	0.5	0.8-2.3	-5	28	-25-31	2	0.0	2-2
**Brainstem**	0.89	0.08	0.78-0.95	0.97	0.08	0.82-1.0	3.0	1.5	1.2-6.0	77	143	-25-251	2	0.0	0-2
**Esophagus**	0.80	0.07	0.71-0.87	0.94	0.05	0.87-1.0	6.0	4.5	1.2-10.1	165	148	-92-332	2	0.3	1-3
**Femur_Head_L**	0.86	0.03	0.81-0.88	0.85	0.07	0.78-0.89	26.7	9.5	15.0-46.5	-1	49	-190-176	2	1.5	0-3
**Femur_Head_R**	0.89	0.07	0.72-0.91	0.89	0.13	0.55-0.93	16.5	6.2	9.0-21.2	-8	38	-460-104	2	1.0	1-3
**Heart**	0.94	0.06	0.66-0.97	0.87	0.14	0.59-0.93	8.2	10.3	3.8-52.5	82	168	-32-246	2	0.3	1-3
**Kidney_L**	0.95	0.08	0.90-1.0	0.97	0.08	0.87-1.0	2.4	5.4	0.0-7.9	25	89	-344-286	3	1.0	1-3
**Kidney_R**	0.93	0.09	0.89-1.0	0.93	0.11	0.85-1.0	3.5	5.9	0.0-8.0	35	146	-55-470	3	1.0	1-3
**Lens_L**	0.76	0.12	0.72-0.89	1.00	0.00	1.0-1.0	1.1	0.8	0.8-3.0	15	9	6-26	3	0.0	2-3
**Lens_R**	0.81	0.14	0.67-0.87	1.00	0.00	0.99-1.0	1.1	0.4	0.8-3.0	18	13	4-54	3	0.0	0-3
**Liver**	0.98	0.05	0.94-1.0	0.98	0.09	0.83-1.0	1.4	4.0	0.0-6.0	197	261	-2-409	2	1.0	1-3
**Lung_L**	0.96	0.02	0.92-0.98	0.92	0.07	0.83-0.98	5.0	2.6	2.5-10.4	2	45	-121-61	3	0.3	1-3
**Lung_R**	0.95	0.03	0.91-0.96	0.86	0.07	0.73-0.91	7.8	5.6	4.5-13.2	10	48	-4-163	3	1.0	2-3
**OpticNrv_L**	0.70	0.21	0.47-0.79	0.91	0.18	0.78-1.0	9.3	12.5	1.2-17.6	-3	36	-25-95	2	0.5	0-3
**OpticNrv_R**	0.64	0.13	0.50-0.80	0.90	0.12	0.77-0.99	8.6	9.8	1.2-21.5	8	61	-26-140	2	0.3	1-3
**Pituitary**	0.75	0.26	0.30-0.89	1.00	0.03	0.75-1.0	1.7	1.7	0.8-4.8	13	16	-3-64	2	1.3	1-3
**Rectum**	0.87	0.12	0.70-0.91	0.87	0.21	0.70-0.97	8.0	23.6	3.0-39.8	130	102	54-212	2	1.1	1-3
**SpinalCanal**	0.82	0.19	0.65-1.0	0.89	0.20	0.72-1.0	70.1	89.0	0.0-153.0	-2	20	-52-69	3	1.0	2-3
**Spleen**	0.92	0.06	0.67-1.0	0.97	0.08	0.43-1.0	3.0	2.2	0.0-16.9	62	131	-234-312	3	1.0	1-3

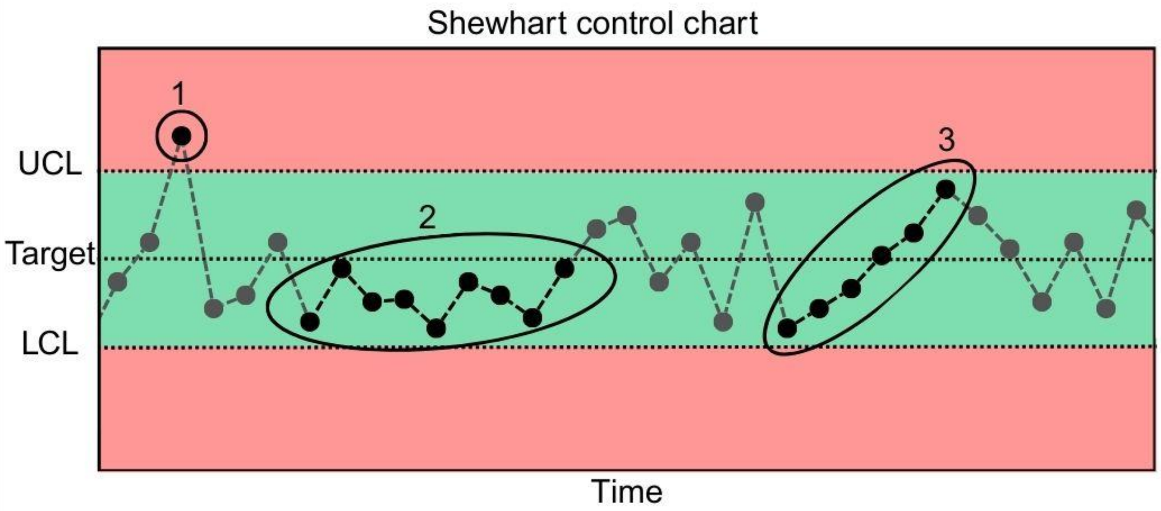

Supplementary Figure S1: A visualisation that shows how the Nelson rules can be detected in a Shewhart control chart. The numbers in this figure correspond to the rules in Table 2.

<u>Supplementary material A</u>

Statistical process control (SPC) is a statistical method used to detect process errors to improve process quality [19]. SPC is established as a standard quality control (QC) method in nonmedical fields, such as manufacturing and processing industries. It makes use of control charts to monitor the process in real-time and process capability indices (PCIs) to quantify the process capability. These capability indices measure the ability of a process to operate within the specification limits. Specification limits are defined limits that are typically set as requirements by the manufacturer or user [19]. As of now, no specification limits are set for the DLS model performance to calculate the PCIs. In the future this can be done to identify areas where improvements may be needed.

Different to specification limits, control limits are not pre-set as they are determined by the data distribution of the real-time output [20]. Control limits are part of the parameters required to create control charts, referred to as the SPC parameters in this article. As mentioned in section 2, typically the target is set at the mean with the lower and upper control limits 3 standard deviation (σ) away from the target. This is done such that there is a 0.135% probability that any data point belonging to a normally distributed population will be outside of the control limits [20].

Control charts are combined with Nelson rules to monitor process control and process stability [21]. In Table S2 the eight Nelson rules are given as they are originally defined and their purpose in typical stable manufacturing processes.

Supplementary Table S2: Nelson test rules. Taken from Muralidharan et al. [20], originally designed by Nelson et al.[21].

Number	Definition	Purpose
Rule 1	One data point outside of the control limit	Detect out-of-control data
Rule 2	Nine consecutive data points on one side of the centre line	Detect a process shift
Rule 3	Six consecutive data points either increasing or decreasing	Detect a process drift
Rule 4	Fourteen consecutive data points alternating up and down	Detect a process alternating between two states
Rule 5	Two out of three data points falling outside of two σ from the centre line	Detect an intermediate shift
Rule 6	Four out of vie data points falling outside of two σ from the centre line	Detect a small shift
Rule 7	Fifteen data points falling within one sigma from the centre line	Detect a reduction in process variability
Rule 8	Eight consecutive datapoints falling outside of one sigma from the centre line	Detect a mixed process behaviour

As noted in Section 2.2, adjustments to standard SPC parameters were made to better accommodate the DLS CQA use case, which differs from typical manufacturing scenarios. A non-normal alternative was introduced for SPC parameters, as normality cannot be assumed for all metrics. The median is used as the target for non-normally distributed data, with control limits set at percentiles corresponding to the probability of a data point falling outside normal distribution limits. Additionally, 2σ was selected instead of 3σ to increase sensitivity, enabling earlier detection of deviations. This choice is particularly relevant, as PCIs, typically used in manufacturing to assess process stability, cannot be calculated in this use case, necessitating more manual investigations to monitor the process at early stages.

Adaptations to Nelson rules, as mentioned in Table 1 in section 2, were required to better fit the use case. With the addition of the voting system in rule 1 it is made sure that an outlier is not detected on only one metric. This helps especially for smaller ROIs which can have a low VDSC, approximately 0.0, but a high SDSC, approximately 1.0. Otherwise, smaller ROIs might be over represented in the outlier set. The other adaptation is made for rule 2. For some ROI-metric combinations the target is too close to its potential limit, for example a VDSC of 1.0 and a HD95 of 0 mm. Without adding the 0.5 % tolerance boundary the second rule will be triggered too often for structures that, on average, receive little adaptations as even the slightest adaptation will push the metric below the target.
